# Neurosurgical intervention versus conservative management in older adults with traumatic brain injury: a protocol for a systematic review and meta-analysis

**DOI:** 10.3389/fneur.2026.1775791

**Published:** 2026-05-19

**Authors:** Ravi Shankar, Yuen Ling Hue, Karen Sui Geok Chua

**Affiliations:** 1 Clinical Research & Innovation Office, Tan Tock Seng Hospital, National Healthcare Group, Singapore, Singapore; 2 Department of Rehabilitation Medicine, Tan Tock Seng Hospital, Singapore, Singapore; 3 Institute of Rehabilitation Excellence, Tan Tock Seng Hospital Rehabilitation Centre, Tan Tock Seng Hospital, Singapore, Singapore; 4 Yong Loo Lin School of Medicine, National University of Singapore, Singapore, Singapore; 5 Lee Kong Chian School of Medicine, Nanyang Technological University, Singapore, Singapore; 6 Rehabilitation Research Institute of Singapore, Nanyang Technological University, Singapore, Singapore

**Keywords:** conservative management, craniotomy, decompressive craniectomy, neurosurgical intervention, older adults, traumatic brain injury

## Abstract

**Background:**

Traumatic brain injury in older adults represents a serious public health challenge with complex management decisions. The aging population exhibits distinct pathophysiological responses to brain injury, altered recovery trajectories, and increased vulnerability to both surgical and medical complications. Decision-making regarding neurosurgical intervention versus conservative management remains contentious, with limited high-quality evidence specific to older populations. Existing guidelines predominantly derive from younger cohorts, potentially misaligning with the unique risk–benefit profiles of older adults.

**Objectives:**

This systematic review and meta-analysis protocol aims to synthesize evidence comparing neurosurgical intervention with conservative management for older adults with traumatic brain injury, examining functional outcomes, mortality, quality of life, and complications to inform evidence-based clinical decision-making.

**Methods:**

Following PRISMA-P guidelines, we will search PubMed, MEDLINE, Embase, Scopus, Cochrane Library, Web of Science, CINAHL, PsycINFO, and PEDro from inception to December 2025. Eligibility criteria include adults aged 65 years and older with traumatic brain injury comparing neurosurgical intervention with conservative management. Two reviewers will independently screen studies and extract data using standardized forms. Risk of bias assessment will employ the Cochrane RoB 2 tool for randomized trials and ROBINS-I for non-randomized studies. Random-effects meta-analysis will be conducted where appropriate, with heterogeneity assessed using I^2^ statistics. Subgroup analyses will explore age strata, injury severity, frailty status, and intervention types. GRADE methodology will evaluate certainty of evidence.

**Discussion:**

This protocol establishes rigorous methodology for synthesizing comparative effectiveness evidence on neurosurgical versus conservative management in older adults with traumatic brain injury. Findings will address critical evidence gaps, informing clinical guidelines, shared decision-making frameworks, and research priorities for optimizing outcomes in this vulnerable population.

**Systematic Review Registration:**

https://www.crd.york.ac.uk/PROSPERO/view/CRD420261376172, identifier PROSPERO (CRD420261376172).

## Introduction

Traumatic brain injury among older adults has emerged as a major public health crisis, with incidence rates tripling over the past two decades as populations age globally (1). Adults aged 65 years and older now represent the demographic with the highest rates of TBI-related hospitalizations and deaths, surpassing rates in younger populations traditionally considered at highest risk (2). Falls are the leading cause of TBI in older adults, accounting for more than 80% of cases, with motor-vehicle accidents and assaults making up much smaller proportions (3–5). The convergence of population aging, increased longevity, higher activity levels among older adults, and widespread anticoagulation use has created an epidemiological shift requiring fundamental reconsideration of TBI management approaches developed primarily from younger cohort evidence.

The pathophysiology of TBI in older adults differs substantially from younger populations due to age-related changes in brain structure and function. Cerebral atrophy increases subdural space, allowing greater brain movement during impact and increasing vulnerability to subdural hematomas even from minor trauma (6). Decreased brain compliance and impaired autoregulation compromise the aged brain’s ability to maintain perfusion during secondary injury cascades. Cellular senescence reduces regenerative capacity while neuroinflammatory responses become dysregulated and prolonged (7). Comorbidities including cardiovascular disease, diabetes, and chronic kidney disease further complicate pathophysiology and recovery. Polypharmacy, particularly anticoagulation and antiplatelet therapy, increases hemorrhage risk and progression. These factors collectively create a distinct clinical entity requiring management approaches specifically validated in older populations rather than extrapolated from younger cohort studies.

The decision between neurosurgical intervention and conservative management for older adults with TBI represents one of the most challenging and consequential in neurosurgery and critical care. Neurosurgical options include craniotomy for hematoma evacuation, decompressive craniectomy for refractory intracranial hypertension, external ventricular drainage for hydrocephalus, and burr hole drainage for chronic subdural hematomas. Conservative management encompasses medical optimization of intracranial pressure, coagulopathy reversal, seizure prophylaxis, and supportive care without surgical intervention. The optimal approach remains highly controversial, with wide practice variation across institutions and countries reflecting uncertainty in the evidence base (8).

Arguments favoring neurosurgical intervention emphasize the potential for rapid decompression preventing secondary brain injury, improved survival in selected cases, and possibility of meaningful recovery despite advanced age. Proponents cite evidence suggesting that chronological age alone should not determine treatment eligibility and that selected older adults can achieve favorable outcomes following surgery (9). Conversely, arguments for conservative management highlight high perioperative mortality and morbidity in older adults, frequent progression to severe disability despite surgical intervention, prolonged recovery trajectories often incompatible with pre-injury quality of life, and resource utilization considerations in healthcare systems with limited capacity (10). The complexity of these considerations is compounded by heterogeneity within older adult populations, where physiological age, frailty, cognitive reserve, and social support may influence outcomes more than chronological age. A landmark position paper by Depreitere et al. (11) highlighted frailty, multimorbidity, and elevated risk of dementia as key drivers of the natural history of TBI in older adults, emphasizing that these factors, rather than chronological age alone, should inform prognosis and treatment decisions. The present systematic review directly addresses this call by examining the weight of these factors in determining outcomes of neurosurgical intervention versus conservative management.

Current clinical guidelines provide limited specific guidance for managing TBI in older adults, with most recommendations extrapolated from general adult populations dominated by younger patients (12). The Brain Trauma Foundation guidelines acknowledge insufficient evidence for age-specific recommendations while noting that older adults consistently demonstrate worse outcomes across all injury severities. This evidence gap has profound implications as clinicians make high-stakes decisions affecting survival, functional independence, and quality of life without robust age-specific evidence. A scoping review by Dasic et al. (13) examining challenges and improvement programs across neurotrauma services explicitly identified the management of elderly patients as a priority area of unmet need, underscoring the urgency of generating high-quality comparative evidence in this population. Families struggle with decision-making absent clear prognostic information relevant to their loved one’s age and circumstances. Healthcare systems allocate resources without understanding comparative effectiveness and cost-effectiveness of different approaches in older populations.

This systematic review and meta-analysis aims to synthesize evidence comparing neurosurgical intervention with conservative management for older adults with traumatic brain injury. The primary objective is to determine the comparative effectiveness of neurosurgical intervention versus conservative management on mortality and functional outcomes in adults aged 65 years and older with traumatic brain injury. Secondary objectives include examining quality of life outcomes, complication rates, and identifying patient characteristics associated with differential treatment responses.

The review addresses several critical research questions essential for clinical decision-making. The primary research question asks: In older adults aged 65 years and older with traumatic brain injury, how does neurosurgical intervention compare with conservative management regarding short-term and long-term mortality? Secondary questions explore: What are the comparative effects on functional independence as measured by validated scales including Glasgow Outcome Scale, Functional Independence Measure, and activities of daily living assessments? How do treatments compare regarding quality of life outcomes for survivors and their caregivers? What are the relative rates of complications including infections, thromboembolic events, and need for institutional care? Which patient characteristics including age strata, frailty status, injury severity, and comorbidity burden predict differential responses to surgical versus conservative management? These questions recognize the multidimensional nature of outcomes important to older adults, extending beyond survival to encompass functional independence, quality of life, and treatment burden considerations.

## Methods

### Study design and protocol registration

This systematic review and meta-analysis protocol adheres to the Preferred Reporting Items for Systematic Review and Meta-Analysis Protocols (PRISMA-P) 2015 statement, ensuring transparent and complete reporting of planned methods (14). The protocol was registered with the International Prospective Register of Systematic Reviews (PROSPERO) before commencing database searches (CRD420261376172). The review will follow Cochrane Collaboration methodology for systematic reviews of interventions, adapted for observational studies which will likely comprise the majority of evidence given ethical constraints on randomizing TBI management (15). Any protocol amendments will be documented with dates, descriptions of changes, and rationales. The review team includes expertise in neurosurgery, geriatric medicine, critical care, systematic review methodology, and biostatistics, ensuring comprehensive perspectives on this complex clinical question.

### Conceptual framework

The PICOS framework (Population, Intervention, Comparator, Outcomes, Study design) structures this systematic review, providing clarity for study selection and analysis planning (16). The Population comprises adults aged 65 years and older with traumatic brain injury of any severity, recognizing that management decisions span from mild complicated TBI with small hemorrhages to severe injuries with mass effect. The Intervention encompasses any neurosurgical procedure performed for TBI management including craniotomy, craniectomy, burr hole drainage, external ventricular drainage, or intracranial pressure monitor placement. The Comparator is conservative or medical management without neurosurgical intervention, including best medical therapy, intensive care support, and comfort care approaches. Outcomes include mortality at multiple timepoints, functional status using validated measures, quality of life assessments, complications, discharge disposition, and resource utilization. Study designs include randomized controlled trials though expected to be rare, prospective and retrospective cohort studies, case–control studies, and large registry analyses providing comparative data.

This framework accommodates the anticipated heterogeneity in TBI populations, interventions, and outcomes while maintaining focus on the fundamental question of surgical versus conservative management. The broad inclusion of TBI severities recognizes that management controversies exist across the spectrum from mild complicated injuries where surgery might be considered for small hemorrhages to severe injuries where futility concerns arise. The framework’s structure enables examination of effect modification by patient and injury characteristics crucial for personalized decision-making.

### Eligibility criteria

Population criteria include adults aged 65 years and older at time of injury with acute traumatic brain injury diagnosed by clinical assessment and neuroimaging. The age threshold of 65 years aligns with common definitions of older adults in TBI literature while recognizing heterogeneity within this population. Studies including mixed age populations are eligible if subgroup data for adults 65 years and older can be extracted or if mean age exceeds 70 years with narrow distribution. All TBI mechanisms are eligible including falls, motor vehicle accidents, assaults, and others, reflecting real-world injury patterns in older adults. Studies must report TBI severity using recognized classifications such as Glasgow Coma Scale, Abbreviated Injury Scale, or radiographic criteria. Exclusion criteria include penetrating brain injury given different pathophysiology and management, non-traumatic intracranial hemorrhage such as spontaneous intracerebral or subarachnoid hemorrhage, and studies exclusively examining chronic phase management beyond acute hospitalization. Studies involving military personnel with combat-related TBI are excluded due to distinct injury mechanisms, patient demographics, and healthcare contexts that limit generalizability to civilian older adult populations.

Intervention criteria require clear documentation of neurosurgical intervention performed for TBI management. Eligible procedures include craniotomy for acute subdural, epidural, or intracerebral hematoma evacuation; decompressive craniectomy for intracranial hypertension or mass effect; burr hole drainage for chronic subdural hematoma; external ventricular drainage for hydrocephalus or intracranial pressure monitoring; and combination procedures. The timing of intervention relative to injury must be reported. Studies where surgical decision-making cannot be determined or where surgery was performed for non-TBI indications are excluded. The comparator must be conservative or medical management without neurosurgical intervention. This includes intensive care management with medical optimization, reversal of anticoagulation, seizure prophylaxis, hyperosmolar therapy, and supportive care. Studies must provide sufficient detail to confirm that comparison groups received contemporary standard care rather than nihilistic withdrawal based solely on age.

Outcome criteria require reporting of at least one primary outcome of mortality or functional status. Mortality must be reported at defined timepoints such as in-hospital, 30-day, 90-day, 6-month, or 1-year. Functional outcomes include Glasgow Outcome Scale or Extended Glasgow Outcome Scale, Functional Independence Measure, Modified Rankin Scale, Barthel Index, or activities of daily living assessments. Secondary outcomes of interest include quality of life measured by validated instruments, cardiovascular outcomes including myocardial infarction and stroke, frailty progression measured by validated frailty indices, infection rates categorized by type and severity, discharge disposition including home versus institutional care, complications such as infections or thromboembolism, hospital and intensive care length of stay, and resource utilization metrics. Studies must report outcomes separately for surgical and conservative management groups with sufficient data for effect size calculation.

Study design criteria include all comparative studies providing data on both neurosurgical and conservative management approaches. Randomized controlled trials are eligible though expected to be rare given ethical considerations. Observational studies including prospective cohorts, retrospective cohorts, case–control studies, and registry-based studies are anticipated to provide most evidence. Studies must include a minimum of 20 patients per treatment group to ensure statistical stability. Case reports, case series without comparison groups, reviews, editorials, and studies published only as abstracts are excluded. No language restrictions apply, with translation arranged for non-English publications. The timeframe encompasses studies from database inception to December 2025, capturing evolution in both neurosurgical techniques and conservative management approaches. Studies conducted exclusively in military or wartime settings are excluded given different triage protocols, resource availability, injury patterns, and patient populations compared to civilian healthcare contexts (see Table 1).

**Table 1:** Inclusion and exclusion criteria.

Criteria	Inclusion	Exclusion
Population	Adults ≥65 years with acute traumatic brain injury; Mixed ages if older adult data extractable	Age <65 years exclusively; Penetrating brain injury; Non-traumatic hemorrhage; Military/combat TBI; War-related injuries
Intervention	Neurosurgical procedures for TBI (craniotomy, craniectomy, burr holes, EVD)	Non-TBI surgery; Unclear surgical indication
Comparator	Conservative/medical management without surgery	No comparison group; Inadequate medical care
Outcomes	Mortality or functional status at defined timepoints; Secondary outcomes (QoL, complications)	No relevant outcomes; Insufficient data for analysis
Study design	RCTs, cohort studies, case–control studies, registries; ≥20 patients per group	Case reports/series; Reviews; Abstracts only
Time period	Database inception to December 2025	None

### Information sources and search strategy

Comprehensive database searching will encompass six major biomedical databases ensuring broad coverage of neurosurgical, geriatric, and critical care literature. Primary databases include MEDLINE via PubMed for comprehensive biomedical coverage, Embase for international literature including European and Asian studies, and Cochrane Central Register of Controlled Trials for randomized evidence. Specialized databases comprise Scopus for broad biomedical and interdisciplinary indexing, Web of Science for citation tracking and multidisciplinary coverage, CINAHL for nursing and allied health perspectives on TBI care, PsycINFO for psychological and neuropsychological outcomes, PEDro for physiotherapy and rehabilitation evidence, and ProQuest for dissertations and grey literature. This combination ensures identification of studies from diverse clinical disciplines involved in older adult TBI management.

The search strategy combines controlled vocabulary and free-text terms across four conceptual domains developed iteratively with a medical librarian. Terms for traumatic brain injury include MeSH/Emtree headings and keywords such as “brain injuries, traumatic,” “craniocerebral trauma,” “head injuries, closed,” “subdural hematoma,” “epidural hematoma,” and “cerebral contusion.” Age-related terms encompass “aged,” “aged, 80 and over,” “geriatrics,” “older adult,” “elderly,” and specific age ranges. Intervention terms include “neurosurgical procedures,” “craniotomy,” “decompressive craniectomy,” “burr hole,” “trephination,” and “surgical decompression.” Comparative terms incorporate “conservative treatment,” “medical management,” “non-operative,” and “comparative effectiveness.” Boolean operators and proximity searching optimize sensitivity while maintaining feasible retrieval numbers.

Supplementary search strategies will identify additional relevant studies through multiple approaches. Citation searching includes screening reference lists of included studies and relevant systematic reviews, forward citation tracking of key studies using Web of Science and Google Scholar. Grey literature searching encompasses ProQuest Dissertations and Theses Global, conference proceedings from neurosurgical and geriatric societies, and clinical trial registries including ClinicalTrials.gov and WHO International Clinical Trials Registry Platform. Content experts in neurosurgery and geriatric trauma will be contacted to identify unpublished or in-press studies. Professional societies including the American Association of Neurological Surgeons, Congress of Neurological Surgeons, and International Neurotrauma Society will be approached for relevant data.

### Study selection

Study selection will employ a two-stage screening process managed through Covidence systematic review software (17). Two reviewers will independently screen titles and abstracts against basic eligibility criteria, with disagreements resolved by consensus or third reviewer adjudication. Full-text screening will assess detailed eligibility with specific reasons for exclusion documented. Calibration exercises using 50 randomly selected citations will ensure consistent criteria application before proceeding with complete screening. Inter-rater agreement will be assessed using Cohen’s kappa statistics with values exceeding 0.75 considered acceptable.

The screening form will be piloted and refined to ensure clear operationalization of eligibility criteria. Particular attention will focus on confirming population age, distinguishing TBI surgery from other neurosurgical procedures, and verifying presence of comparison groups. Studies with unclear eligibility will prompt author contact for clarification, with three attempts over 4 weeks before exclusion. Non-English studies meeting initial screening will undergo professional translation for full eligibility assessment. A PRISMA flow diagram will document the selection process including numbers screened, excluded, and reasons for exclusion at each stage.

### Data extraction

Data extraction will utilize standardized forms developed specifically for this review and piloted on five diverse studies. Two reviewers will independently extract data with discrepancies resolved through discussion and verification against source documents. The extraction form captures study characteristics including authors, publication year, country, setting, study design, funding sources, and conflicts of interest. Population characteristics encompass sample size, age distribution, sex, race/ethnicity, mechanism of injury, TBI severity measures, Glasgow Coma Scale scores, radiographic findings, comorbidities, frailty assessments, anticoagulation status, and time from injury to presentation.

Intervention details include type of neurosurgical procedure, timing relative to injury, surgeon experience or hospital volume, perioperative management protocols, and any repeat surgeries. Conservative management details encompass specific medical treatments provided, intensive care protocols, intracranial pressure management strategies, and criteria for withholding or withdrawing life-sustaining treatment. Data extraction will specifically document whether conservative management groups included patients for whom life-sustaining treatment was withdrawn or withheld based on perceived poor prognosis, as this represents a critical source of potential bias when comparing outcomes between surgical and conservative cohorts. Studies will be categorized by whether they explicitly excluded patients with care limitation orders, included such patients with documentation of decision-making criteria, or did not report on care limitation practices. Outcome data extraction includes mortality at all reported timepoints with causes of death when available, functional outcome scores with instruments used and assessment timing, quality of life measures for patients and caregivers, complications categorized by system and severity, discharge disposition and care needs, readmission rates, and resource utilization including length of stay and costs. For mortality outcomes, time from injury to death will be extracted when reported to enable survival analysis. Regarding interventions, middle meningeal artery embolization will be classified as a minimally invasive intervention separate from traditional neurosurgical procedures, given its distinct risk profile and technical approach. Studies employing this technique will be analyzed as a separate subgroup to assess comparative effectiveness of this emerging intervention.

Methodological features extracted include study design specifics, sampling and recruitment methods, inclusion and exclusion criteria, handling of missing data, statistical methods including adjustment for confounding, and subgroup analyses performed. For adjusted analyses, variables included in multivariable models will be recorded. Particular attention will be paid to extraction of frailty measures including Clinical Frailty Scale scores, Frailty Index values, or other validated frailty assessment tools, given the anticipated role of frailty as a key effect modifier in treatment response among older adults with TBI. Authors will be contacted to obtain missing key data or clarify methodological details, with all correspondence documented. Data from multiple reports of the same study will be collated to maximize information while avoiding duplication.

### Risk of bias assessment

Risk of bias assessment will employ tools appropriate for different study designs. For the rare randomized controlled trials, the Cochrane Risk of Bias 2 (RoB 2) tool will evaluate bias arising from the randomization process, deviations from intended interventions, missing outcome data, measurement of outcomes, and selection of reported results (18). Each domain receives judgment of low risk, some concerns, or high risk, with overall risk determined by the highest risk domain. For observational studies comprising most anticipated evidence, the Risk of Bias in Non-randomized Studies of Interventions (ROBINS-I) tool will assess bias due to confounding, selection of participants, classification of interventions, deviations from intended interventions, missing data, measurement of outcomes, and selection of reported results (19).

Particular attention will focus on confounding by indication, where patients receiving surgery may systematically differ from those managed conservatively in ways affecting outcomes beyond measured variables. Studies will be evaluated for appropriate adjustment methods including multivariable regression, propensity score matching, or instrumental variable analysis. The quality of mortality ascertainment will be assessed given potential for differential follow-up between treatment groups. Functional outcome assessment quality will consider blinding of assessors, standardization of instruments, and timing of assessments. Two reviewers will independently perform bias assessments with disagreements resolved through discussion. Risk of bias will inform sensitivity analyses and GRADE certainty assessments rather than determining study exclusion.

### Data synthesis

Data synthesis will begin with narrative description of included studies examining populations, interventions, and outcomes studied. Descriptive statistics will summarize study characteristics including geographic distribution, publication years, sample sizes, and methodological features. Clinical heterogeneity will be assessed by examining variation in populations such as TBI severity distribution, intervention characteristics including surgical techniques and timing, and outcome assessment methods.

Meta-analysis will be conducted where sufficient homogeneity exists in populations, interventions, and outcomes. Effect measures will include risk ratios for dichotomous outcomes such as mortality and unfavorable functional outcome, mean differences for continuous outcomes such as length of stay, and hazard ratios for time-to-event outcomes where available. Random-effects models using restricted maximum likelihood estimation will be employed given expected heterogeneity across studies. Statistical heterogeneity will be quantified using *I*^2^ statistics, with values of 25%, 50%, and 75% representing low, moderate, and high heterogeneity, respectively. Tau-squared will estimate between-study variance. Forest plots will display individual study effects and pooled estimates with 95% confidence intervals.

Where meta-analysis proves inappropriate due to excessive heterogeneity or incompatible outcome reporting, narrative synthesis will examine patterns across studies. Vote counting based on direction of effect will assess consistency of findings. Harvest plots may visualize patterns in effects across different outcomes and study characteristics. Effect sizes will be presented in summary tables even when statistical pooling is not performed. Dose–response relationships will be explored where studies report outcomes across age strata or injury severity gradients.

Subgroup analyses will explore sources of heterogeneity and identify potential effect modifiers. Planned subgroups include age categories comparing 65–74, 75–84, and 85+ years; TBI severity comparing mild, moderate, and severe injuries; specific TBI types such as subdural versus epidural hematomas; timing of surgery comparing ultra-early, early, and delayed intervention; frailty status where reported using validated instruments; and study quality comparing low versus high risk of bias studies. Additional subgroup analyses will examine cardiovascular complications comparing rates of myocardial infarction, cardiac arrhythmias, and cerebrovascular events between surgical and conservative management groups. Infection-related subgroups will stratify surgical site infections, pneumonia, urinary tract infections, and sepsis to identify differential risks by management approach. These analyses recognize that competing causes of morbidity and mortality may differ substantially between treatment groups and across age strata. Meta-regression will examine continuous moderators including mean age, injury severity scores, and publication year. Sufficient studies per subgroup (minimum 10) will be required for robust analysis.

Sensitivity analyses will examine the robustness of findings to analytical decisions. Analyses will compare fixed versus random effects models, exclude studies with high risk of bias, exclude outlier studies identified through influence analysis, compare adjusted versus unadjusted effect estimates, and examine impact of missing data assumptions. Publication bias assessment will employ funnel plots for visual inspection where at least 10 studies are available, Egger’s test for funnel plot asymmetry, and trim-and-fill analysis to estimate impact of potentially missing studies. However, interpretation will acknowledge that asymmetry may reflect heterogeneity rather than publication bias in observational studies.

### Certainty of evidence assessment

The Grading of Recommendations Assessment, Development and Evaluation (GRADE) approach will evaluate certainty of evidence for each outcome (20). Initial certainty ratings will reflect study designs, with randomized trials starting as high certainty and observational studies as low certainty. Certainty may be downgraded for risk of bias if most evidence comes from studies with serious limitations, inconsistency if substantial unexplained heterogeneity exists, indirectness if populations or interventions differ from those of interest, imprecision if confidence intervals include both benefit and harm, and publication bias if evidence suggests missing studies. Certainty may be upgraded for large magnitude of effect, dose–response gradient, or when plausible confounding would reduce demonstrated effect. Final certainty ratings of high, moderate, low, or very low will indicate confidence that true effects lie close to estimated effects.

## Discussion

This systematic review protocol establishes comprehensive methodology for synthesizing evidence comparing neurosurgical intervention with conservative management in older adults with traumatic brain injury. The review addresses a critical evidence gap with profound implications for clinical practice, healthcare policy, and research priorities in the context of global population aging. The protocol’s strength lies in its specific focus on older adults, recognizing that evidence from younger populations cannot be directly extrapolated given distinct pathophysiology, recovery patterns, and treatment goals in advanced age.

The review occurs at a crucial juncture as healthcare systems worldwide grapple with rising TBI incidence in older adults while facing resource constraints and uncertain evidence for guiding management. The COVID-19 pandemic has heightened awareness of age-based treatment allocation decisions while simultaneously demonstrating that chronological age alone poorly predicts individual outcomes (21). Advances in neurosurgical techniques including minimally invasive approaches and improved perioperative care have reduced surgical risks, potentially shifting risk–benefit calculations. Concurrently, enhanced understanding of frailty, cognitive reserve, and biological aging offers opportunities for more personalized decision-making beyond chronological age. This review will synthesize evidence across these evolving contexts to provide contemporary guidance for clinical practice.

Several anticipated challenges require careful consideration during review conduct. The predominance of observational studies reflects ethical constraints on randomizing TBI management but introduces substantial risk of confounding by indication. Patients selected for surgery likely differ systematically from those managed conservatively in measured and unmeasured ways affecting outcomes. While statistical adjustment and propensity score methods partially address this, residual confounding remains probable. The review will carefully assess and report these limitations while extracting maximum insight from available evidence. Sensitivity analyses comparing crude and adjusted estimates will explore confounding impact.

Heterogeneity across multiple dimensions poses analytical challenges. TBI severity ranges from mild complicated injuries with small hemorrhages to devastating injuries with brainstem compression. Surgical interventions vary from minimally invasive burr holes to extensive decompressive craniectomies. Conservative management spans from aggressive medical optimization to comfort-focused care. Outcomes important to older adults extend beyond survival to functional independence, cognitive preservation, and quality of life. The review will embrace this complexity through comprehensive subgroup analyses while acknowledging that some questions may require individual patient data meta-analysis for definitive answers.

The definition and measurement of outcomes in older adults requires careful consideration. Traditional dichotomization of outcomes as favorable versus unfavorable using Glasgow Outcome Scale may inadequately capture meaningful differences for older adults. Return to pre-injury function rather than absolute functional level may better reflect successful outcomes. Quality of life measures validated in older populations are essential given that survival with severe disability may not align with individual values and preferences. The review will examine multiple outcome dimensions while acknowledging measurement challenges in this population including high rates of pre-existing cognitive impairment and functional limitations.

Findings will have immediate clinical applications for bedside decision-making. Evidence synthesis will help clinicians and families understand probable outcomes with different management approaches based on patient characteristics. Identification of factors predicting differential treatment response will support personalized decision-making. For example, if evidence suggests that adults aged 65–75 years with moderate TBI benefit from surgery while those over 85 years do not, this would directly inform clinical counseling. Understanding outcome trajectories will facilitate realistic goal-setting and care planning. The review will generate evidence for clinical decision support tools integrating multiple prognostic factors.

Healthcare system implications include informing resource allocation and care pathway development. Understanding comparative effectiveness enables appropriate triage and referral patterns between hospitals with and without neurosurgical capability. Evidence about futility of intervention in specific subgroups could reduce inappropriate transfers and procedures while ensuring access for those likely to benefit. Economic analyses of cost-effectiveness will inform coverage decisions and quality metrics. The review may identify opportunities for improving conservative management protocols that narrow outcome gaps with surgical intervention.

Research priorities emerging from the review will guide future investigations addressing remaining uncertainties. Methodological insights about confounding and bias in observational TBI studies will inform design of future research including potential randomized trials in clinical equipoise zones. Identification of outcome measures most relevant to older adults will standardize future studies enabling better evidence synthesis. Understanding of effect modifiers will guide stratified trial designs and precision medicine approaches. The review may reveal need for studies in underrepresented populations including the oldest old, minority populations, and those with multimorbidity.

Ethical implications of the review extend beyond clinical decision-making to broader questions of healthcare justice and age-based discrimination. Evidence synthesis will examine whether age-based treatment limitations reflect evidence-based futility or ageist assumptions. The review will carefully present evidence about age effects while emphasizing heterogeneity within age groups and importance of individual assessment. Findings will inform development of ethical frameworks balancing individual autonomy, beneficence, non-maleficence, and justice in TBI care for older adults.

Several limitations merit acknowledgment. Language restrictions were not applied, but practical constraints may limit inclusion of non-English studies without available translation. The focus on acute management may miss important evidence about longer-term outcomes and recovery trajectories that influence acute decision-making. Exclusion of studies with fewer than 20 patients per group may eliminate valuable evidence from rare subgroups or specialized interventions. The broad inclusion criteria encompassing heterogeneous populations and interventions may limit definitive conclusions for specific clinical scenarios. These limitations will be transparently reported with implications for evidence interpretation discussed.

This protocol establishes rigorous methodology for synthesizing comparative effectiveness evidence crucial for optimizing TBI management in older adults. The comprehensive approach ensures capture of diverse evidence while maintaining quality standards through systematic bias assessment and GRADE evaluation. The review will generate essential evidence for clinical guidelines, shared decision-making tools, and research priorities in this rapidly evolving field. Ultimately, by clarifying benefits and risks of neurosurgical intervention versus conservative management across the spectrum of older adults with TBI, this synthesis will support treatment decisions that align with individual goals and values while optimizing resource utilization in healthcare systems serving aging populations.
